# Study on the Self-Organization of an Fe-Mn-C-B Coating during Friction with Surface-Active Lubricant

**DOI:** 10.3390/ma13133025

**Published:** 2020-07-06

**Authors:** Marcin Barszcz, Mykhaylo Pashechko, Krzysztof Dziedzic, Jerzy Jozwik

**Affiliations:** 1 Department of Computer Science, Electrical Engineering and Computer Science Faculty, Lublin University of Technology, 36B Nadbystrzycka Street, 20-618 Lublin, Poland; m.barszcz@pollub.pl (M.B.); k.dziedzic@pollub.pl (K.D.); 2 Department of Fundamental of Technology, Fundamentals of Technology Faculty, Lublin University of Technology, 38 Nadbystrzycka Street, 20-618 Lublin, Poland; mpashechko@hotmail.com; 3 Department of Production Engineering, Mechanical Engineering Faculty, Lublin University of Technology, 36 Nadbystrzycka Street, 20-618 Lublin, Poland

**Keywords:** self-organization, friction, wear, coatings, surface-active lubricant

## Abstract

This paper investigates the friction process between an Fe-based coating and C45 steel with surface-active lubrication, as well as examines the coating surface before and after tribological testing. As a result, it is possible to determine whether the surface undergoes self-organization during friction. Coatings were produced by hardfacing a subeutectic alloy Fe-Mn-C-B modified by silicon, nickel, chromium and copper. Tribological tests were performed using a pin-on-disc tribometer. The pin (coating) and the disc made of steel C45 were subjected to heat treatment (hardening and tempering). The tests were carried out under loads of 3 MPa, 7 MPa and 10 MPa at a constant sliding velocity of 0.4 m/s and a sliding distance of 5700 m using a surface-active lubricant (glycerine oil). Obtained results were compared with the published results of previous tests carried out under the same conditions but under a load of 20 MPa. Obtained microscopic and spectroscopic results demonstrate that that the friction pair materials (the coating made of subeutectic alloy Fe-Mn-C-B modified by Si, Ni, Cr, Cu and C45 steel) and the surface-active lubricant cause self-organization during friction. The friction surface of the coatings has a flay-laminar structure and is covered with triboreaction products. The surface shows the presence of wear-resistant compounds such as oxides, carbides, borides and nitrides.

## 1. Introduction

The durability of friction-mating machine elements can be improved by using new or existing solutions regarding design, materials and maintenance in order to significantly reduce friction and wear [1,2,3]. Undoubtedly, one such solution is to create conditions inducing self-organization during friction. According to the current state of the art, self-organization leads to a significant improvement of the tribological properties of friction pairs and wear reduction [4]. The phenomenon of self-organization in friction pairs was identified in the 1970s. Since then, studies and experiments have been conducted to explain this phenomenon. Despite the passing of over 50 years and the growing number of recent scientific publications on the self-organization of tribological systems [5,6,7,8,9,10], this phenomenon has not been yet thoroughly examined. There is no exhaustive definition of the phenomenon either.

Scientists who have attempted to explain the phenomenon of self-organization during friction can be divided into several groups. The first group includes Bershadsky and Kostecki [11]. They believed that so-called secondary structures, i.e., chemical compounds with non-stoichiometric composition, are formed during friction. The authors observed that the properties of these structures such as hardness, chemical composition, phase composition, shape, dimensions and distribution vary depending on friction conditions, specifically the type of material of a friction pair, the type and amount of load, and the type of lubricant [12]. The second group includes I.S. Gershman and N. Bushe [13,14,15,16]. According to these authors, self-organization also protects the friction pair surface from direct contact. This is possibly due to its effect on the secondary structures formed on the surface. A similar observation was made by G. S. Fox-Rabinovich [17,18,19,20,21]. In contrast, D. N. Garkunov and A. Polyakov [22,23] describe the phenomenon of “selective transfer” (as a solution for friction surface self-organization). This phenomenon involves the formation of tribo-coatings as a result of chemical reactions that, in turn, are caused by friction processes. As a result, there occurs a dynamic balance between protective coating formation and wear.

In recent years, scientists have been making additional attempts to explain the phenomenon of self-organization. For example, I. Gershman et al. [5,6] presented the definition of self-organization and described the conditions affecting the occurrence of this phenomenon during friction. They gave examples of the application of this phenomenon in sliding friction pairs. They considered the phenomenon of self-organization and its impact on the friction process, supporting their considerations with various examples and an extensive literature review. They suggested that self-organization promotes the development of wear-resistant materials. They also observed that it leads to reduced wear rate as well as lower friction resistance. This effect was observed during the friction of conductive materials lubricated with natural graphite and during the friction of slide bearings made of graphite alloy sliding materials. They demonstrated that under self-organization, the coefficient of friction could decrease even with increasing unit pressures. The authors of [7] investigated a two-phase copper–silver alloy Cu90-Ag10 under dry friction conditions. Bronze (Cu-Ni-Sn) and stainless steel (SS440C) were used as counterbody materials. It was observed that self-organization also occurs during friction. In both cases, the self-organization was associated with the formation of wear-reducing nanolayers (thin layers). As a result, the wear of SS440C steel surfaces was reduced by 2 times while that of the Cu-Ni-Sn bronze surfaces was decreased by 16 times.

The authors of [8] investigated the effect of various lubricants on the friction of coatings (made of a subeutectic iron-based alloy modified by various elements such as manganese, carbon, boron, silicon, nickel, chromium, copper) and steel C45. Based on obtained results, they chose a lubricant that ensures low wear intensity. They have found that the self-organization of a tribological system is caused by the friction pair material and the surface-active lubricant. The authors of [9] also investigated self-organization of a friction pair using a lubricant. It has been observed that self-organization is caused by surface tension formed at the phase boundary. Nosonovsky in [10] stressed the fact that self-organization contributes to the development of self-lubricating and self-regenerating materials, which has a positive effect on environmental protection. He presented and discussed various models of self-organization during friction.

Summing up, self-organization during friction affects the formation of new thin films directly during operation. So-called operating surface layers are formed; these layers are more susceptible to shear and separate the friction mating elements. They reduce friction resistance and wear and prevent galling. Thus, these layers have a beneficial effect on tribological systems. A good example of operational surface layers exhibiting self-organization during friction are joints of living organisms. They show practically no signs of wear throughout their entire service life and are operated at low friction resistances at the same time. It is possible thanks to constant renewal of protective layers [24,25]. The knowledge and understanding of surface self-organization during friction will definitely accelerate and contribute to the development of new self-lubricating, self-regenerating, self-cleaning and wear-free materials [10]. Therefore, it is purposeful and important to conduct research in this area.

The authors of this paper have undertaken investigations of self-organization during friction relatively recently. They presented their results in [8]. Due to the positive reviews, further work was undertaken in this area. This study is a continuation of the research described in [8] where the impact of lubricants on self-organization was evaluated. This paper presents the results of an investigation of the relationship between changing tribological system loading and the self-organization of an Fe-Mn-C-B-Si-Ni-Cr-Cu coating with low-alloy steel C45 with a surface-active lubricant.

## 2. Materials and Methods

**Figure 1:**
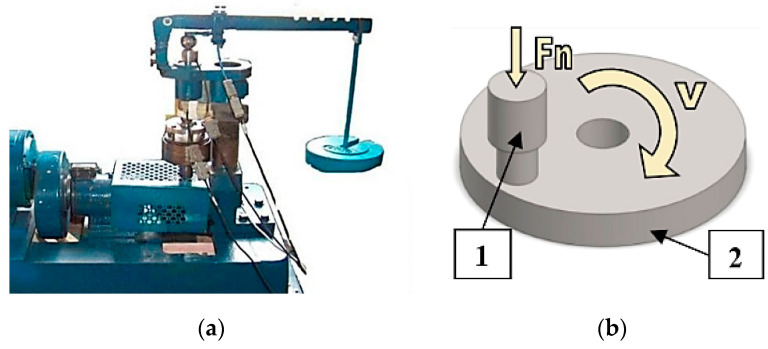
View of (**a**) modernized Amsler tribometer; (**b**) friction pair; 1—sample with a coating (pin), 2—counterbody (disk).

The study of self-organization during friction involved carrying out tribological tests. The tests were performed using a modernized Amsler tribometer (Lublin University of Technology, Lublin, Poland)—Figure 1a. The modernization was made to adapt the test stand to the possibility of conducting tests in compliance with ASTM G133 and G-99. In effect, tribological testing can be carried out in a pin-on-disk or ball-on-disk system with the use of various friction materials, lubricants and unit pressures. Depending on the need, the system can be inverted, i.e., the sample can be used as a pin or a disk. The test stand enables continuous measurement of friction pair temperature and friction force.

Samples and counterbodies were prepared for testing. The sample was a pin made of a steel S235 rod. Coatings were applied to the end face of the samples by gas metal arc welding GMA (MAG with the addition of CO_2_) with the use of flux-cored wires. The core (powder) was made—according to the authors’ recipe—of an eutectic iron-based alloy Fe-Mn-C-B modified by chromium, nickel, silicone and copper. These alloys are highly resistant to abrasive wear. Results of studies investigating the wear resistance of coatings made of these alloys modified by various additives in different configurations and chemical compositions are reported in numerous papers, among others in [8,26,27,28,29,30,31,32]. According to the literature data, copper was added in order to create conditions for self-organization during the friction process. Hardfacing involved using 1.2 mm diameter flux-cored wires with a powder filling of approx. 33%. Hardfacing was performed with an ESAB POWER COMPACT 250 welder (Esab, Goteborg, Sweden). The process was performed with 230 A current and a wire speed of 7 m/min. Padding weld was applied spirally from the centre (two layers were applied). The chemical composition of coatings is given in Table 1. After hardfacing, the pins were machined by turning until the desired shape was obtained (Figure 1b). The pin diameter in the contact zone was 7 mm and the profile roughness parameter was Ra ≈ 1.3 µm. Obtained coatings had a thickness of 2–3 mm and a hardness of 98 HRB. Counterbodies were cut from a C45 steel rod with a diameter of 60 mm. After that, they were machined by turning and grinding until their surface roughness was Ra ≈ 0.5 µm. The shape of a counterbody (55 mm in diameter and 7 mm in thickness) is shown in Figure 1b. The counterbodies were subjected to heat treatment (hardening and tempering). The counterbodies had a hardness of 52 54 HRC. The chemical composition of the C45 steel is given in Table 1.

Tribological tests were performed in a pin-on-disk system. The pin was a fixed sample and the disk was a rotating counterbody. The tests were carried out with a sliding speed of 0.4 m/s and under two friction pair loads: 3 MPa, 7 MPa, 10 MPa and 20 MPa. The friction pair was lubricated by immersion with a surface-active lubricant (glycerine oil; its physical and chemical properties are given in Table 2). The friction time was set equal to 6 h and the sliding distance was 5700 m. The tests were carried out at room temperature. They were repeated 3 times. During the tribological tests, the mass loss of the samples and counterbodies was measured by the weight method. Mass was measured using an Ohaus Discovery (Ohaus, Parsippany, NJ, USA) digital analytical balance (measuring accuracy ± 0.01 mg) every specified time. At the beginning of friction (lapping), mass measurements were made more frequently. Measurements were made after 15, 30, 60, 90, 120, 180, 240, 300 and 360 min. Each time the friction pair was disassembled and then mounted in the same position. This was possible due to the use of appropriate markers. Before weighing, the samples and counterbodies were washed with gasoline and then dried.

The samples and counterbodies were subjected to profilometric testing, microscopic examination and analysis of composition and chemical compounds. Surface roughness evaluation was made using a Surtronic 3+ (Taylor Hobson, Leicester, UK) contact profilometer equipped with a diamond stylus. Measurements were made over a measuring length of 4 mm. They were made perpendicularly to machining traces before tribological tests and perpendicularly to friction traces after tribological tests.

**Figure 2:**
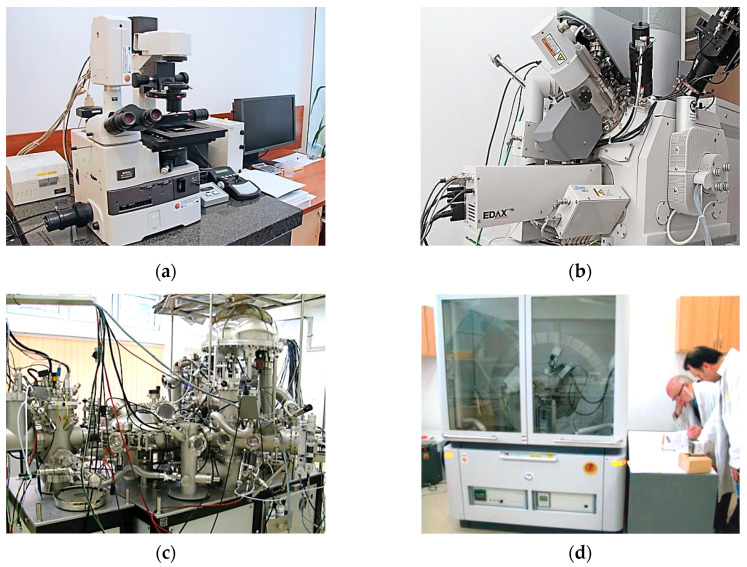
View (**a**) Nikon Eclipse MA 200 metallurgical microscope; (**b**) FEI Quanta 3D FEG scanning electron microscope; (**c**) multi-chamber ultra-high vacuum (UHV) analytical system (Prevac); (**d**) Empyrean high-resolution X-ray diffractometer (Panalytica).

The microstructure of the hardfaced coatings was evaluated using a Nikon Eclipse MA 200 metallurgical microscope (Nikon, Minato, Tokyo, Japan). To make the microstructure visible, the samples prepared for tribological testing were cut along the axis of symmetry. After that, the surfaces (metallographic sections) were ground using sandpaper of different gradations and polished in an aqueous suspension of aluminium oxide. The surface was etched with a 5% solution of nitric acid HNO_3_ in ethanol C_2_H_5_OH. The Nikon Eclipse MA 200 microscope (Figure 2a) was also used to examine the surface of the samples and the counterbodies (before and after friction).

Surface morphology examination at the nanometre scale was performed using a FEI Quanta 3D FEG scanning electron microscope (FEI, Hillsboro, OR, USA)—Figure 2b. Equipped with two SEM/FIB columns: one electron and one ion with a focused ion beam (FIB) setup emitting a gallium ion beam. The electron column was used for scanning. Imaging was performed in a high vacuum mode (<6⋅10^4^ Pa). An Everhadt–Thornley secondary electron detector (ET SED) was used for imaging. Surface examination was carried out at different magnifications to determine its structure. The samples and counterbodies from which specified fragments had been cut were examined. A coated pin was cut from the sample, which resulted in a cylinder with a diameter of 7 mm and a height of 4 mm. An 8 × 8 × 7 mm cube was cut from the counterbody.

The qualitative and quantitative chemical composition of the surface and surface layer and their chemical compounds were determined by X-ray photoelectron spectroscopy (XPS). A multi-chamber ultra-high vacuum (UHV) analytical system (Prevac Rogów, Poland) was used (Figure 2c). The XPS analysis was performed on the samples and counterbodies that were prepared in the same way as those for SEM examination. The examination was carried out on the surface and at a distance (depth) of 10 nm from the surface with the chamber pressure set equal to ~5 × 10^−9^ mbar. Layer etching (removal) was carried out using Ar + ions with an energy of 2 kV and a chamber pressure of ~5 × 10^−6^ mbar. Two types of photoelectron spectra were captured on the surface and the tested depth: overview and detailed spectra of the elements. On this basis, information was obtained regarding the chemical structure of individual elements in the surface layer. Probable chemical structures were determined based on the measured binding energies using literature data [33,34].

The phase composition was determined using an Empyrean high-resolution X-ray diffractometer (Panalytica, Warszawa, Poland)—Figure 2d. Samples were prepared in the same manner as those for X-ray photoelectron X-ray spectroscopy (XPS). The analysis was carried out by the scanning method in a range of 20 = 5–110° (scanning step: 0.05, point measurement time: 3–5 s).

## 3. Results and Discussion

**Figure 3:**
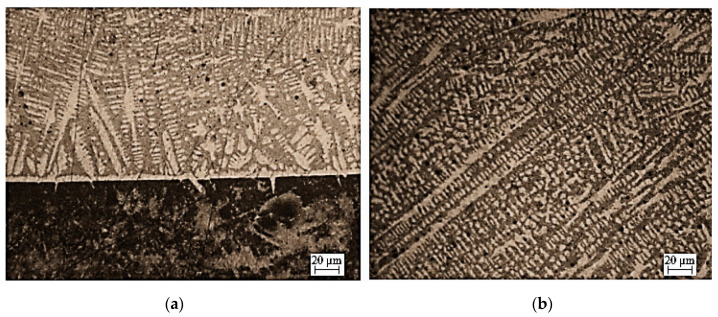
Microstructure of: (**a**) coating-substrate, (**b**) coating made of Fe-Mn-C-B alloy modified by silicon, nickel, chromium and copper.

The structure of the coating (Fe-Mn-C-B alloy modified by silicon, nickel, chromium and copper) is dendritic (Figure 3). Dendrites are formed during hardfacing. It can be observed that they are of different shapes and sizes. They are crystallized in different directions, i.e., perpendicularly and at different angles to the surface of the substrate material. Most of them have short axes, only a few have long axes that are parallel to each other. They do not form any branches.

The phase composition of the eutectic alloy coating is as follows: alloy austenite—soft phase, manganese iron carbide Fe_0.4_Mn_3.6_C—reinforcing phase, and small amounts of dispersed inclusions of iron boride Fe_2_B, chromium boride Cr_2_B, chromium silicide CrSi_2_, chromium carbide Cr_7_C_3_, iron carbide Fe_3_C and copper Cu—dispersed phases.

**Figure 4:**
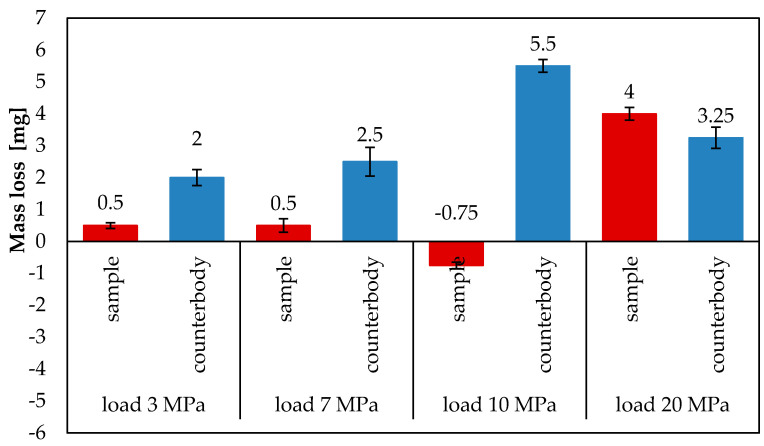
Average mass loss of friction pair materials in tribological tests conducted under 3 MPa, 7 MPa, 10 MPa and 20 MPa [8] and with the use of lubricant (glycerine oil).

The tribological tests made it possible to determine mass loss and friction coefficient. The average mass loss of the Fe-Mn-CB-Si-Ni-Cr-Cu subeutectic alloy and steel C45 after 6 h of tribological testing under sliding friction conditions with the friction node lubricated with a surface-active lubricant (glycerine oil) and loaded with 10 MPa and 20 MPa, is shown in Figure 4. The average mass loss of the coatings under 20 MPa is 4 mg, while that of the counterbodies is 3.25 mg [8]. Under 10 MPa, however, the coating exhibits no mass loss; on the contrary, its mass increases by 0.75 mg. In turn, the average mass loss of the counterbodies is equal to 5.5 mg. At lower loads, the average mass loss of the sample is relatively small and amounts to 0.5 mg for both 7 MPa and 3 MPa. The average mass loss of the counterbody is higher and amounts to 2.5 mg at 7 MPa and to 2 mg at 3 MPa.

**Figure 5:**
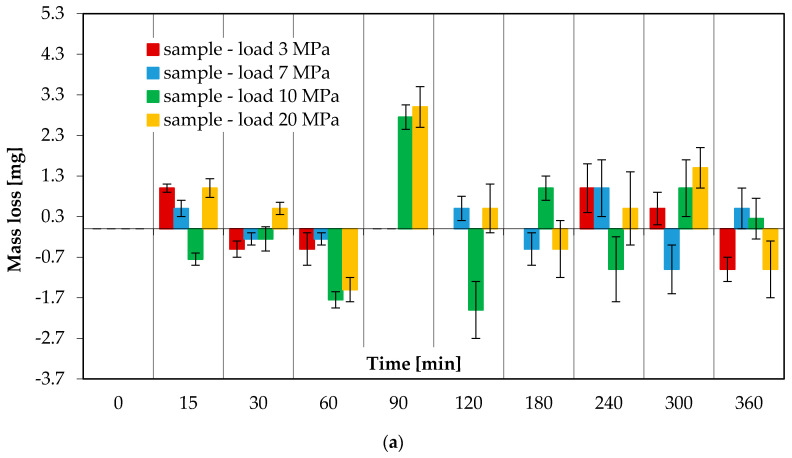

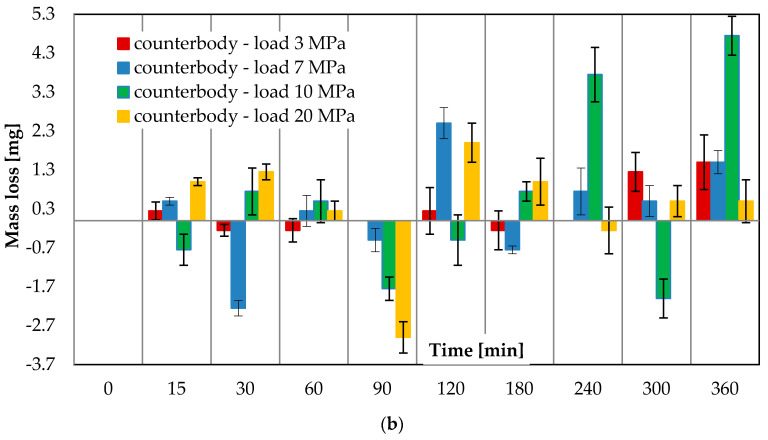
Mass loss of (**a**) samples and (**b**) counterbodies versus friction time under 3 MPa, 7 MPa, 10 MPa and 20 MPa and with glycerine oil lubrication.

Figure 5 shows the mass loss versus friction time. The wear curves of both coated samples and C45 steel counterbodies are taken into account. The characteristics will allow better visualization of the behaviour of the friction pair materials. Figure 5 shows the relationship between mass loss and friction time for a friction pair lubricated with a surface-active lubricant (glycerine oil) and subjected to loads: 3, 7, 10 and 20 MPa.

The results (Figure 5a,b) demonstrate that the mass of the analysed tribological pairs increases and decreases during the entire friction process. The largest mass increase in the samples loaded with 10 MPa occurs after 60 and 120 min of friction (−1.75 mg and −2 mg, respectively), while for those loaded with 20 MPa—after 60 and 360 min of friction, amounting to −1.5 mg and −1 mg, respectively. In turn, the highest mass loss of the samples loaded with both 10 MPa and 20 MPa occurs after 90 min of friction. After 15, 30, 120, 180, 240 and 360 min of friction, the samples loaded with 10 MPa exhibit opposite trends in mass change in relation to those observed under 20 MPa. If there is a mass increase under 10 MPa, there occurs a mass loss under 20 MPa. The largest mass increase in the counterbodies under both 10 MPa and 20 MPa occurs after 90 min of friction (−1.75 mg and −3 mg, respectively). The largest mass loss of the counterbodies under the load of 10 MPa is observed after 240 and 360 min of friction. Similar relationships regarding material transport between the sample and the counterbody during the tribological test can also be observed at the lower loads of 3 MPa and 7 MPa. Obtained values are relatively lower than those obtained at higher loads. In addition to that, no mass loss can be more frequently observed relative to measurement intervals.

**Figure 6:**
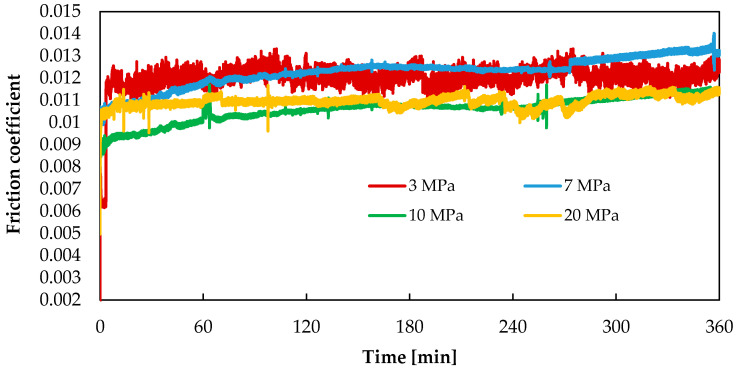
Variations in the coefficient of friction under 3 MPa, 7 MPa, 10 MPa and 20 MPa.

The mass increase and loss of the mating elements may be associated with the migration of wear products and their application to the abrasive surfaces. The migration of components in the friction pair occurs in the lubricant. This contributes to the formation of a thin layer (film) with specific properties on the friction surface. This layer protects the mating surfaces from direct contact. In this way, it helps reduce the rate of material wear, as confirmed by the test results presented in Figure 4. The layer also reduces friction resistance, which is manifested in low values of the friction coefficient. In the friction pairs under study, the coefficient of friction (Figure 6) is 0.011 under 20 MPa, while under 10 MPa it is slightly lower at an early stage of the test, later on in the test reaching a similar value as that obtained for the load of 20 MPa. At lower loads (3 MPa and 7 MPa), the coefficient of friction is slightly higher and amounts to 0.012. It can be observed for all tested loads that the coefficient of friction initially increases and then becomes stable.

The above effects can be explained by the fact that the friction between the copper-containing alloy surface and steel with lubrication (in this case, glycerine) may generate an electrostatic field. Such a field is created as a result of the triboelectric effect and it overlaps with an electrostatic field that is created due to the formation of a double electric layer on the copper-containing alloy surface. At the same time, due to chemisorption as well as mechanical and chemical reactions, new surface-active substances are created and they form a stable disperse system. Copper corrosion products regenerate, while metals and their chemical compounds dissolve in the worn-out glycerine with the occurrence of appropriate ions. As a result, copper-based micelles are formed, with their particle size being approx. 10 nm, which corresponds to colloidal dispersion. The generated electrostatic field ensures the oriented motion of micelles in glycerine due to a difference in the potentials of the steel and copper-containing surfaces. At the beginning of the friction process when copper is positively charged, the micelles decompose on the surface of the cooper-containing alloy. After that, re-charging occurs and a similar process can be observed on the surface of steel, which leads to the formation of a protective layer in the friction zone.

**Figure 7:**
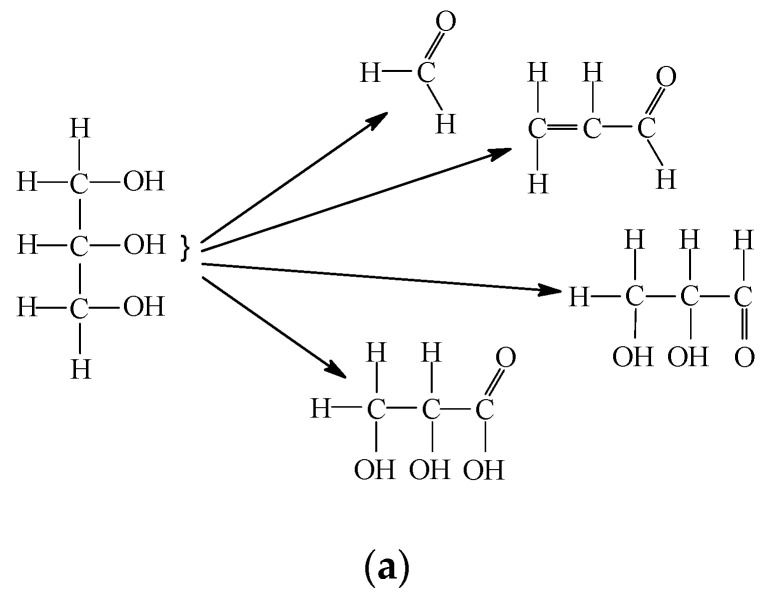

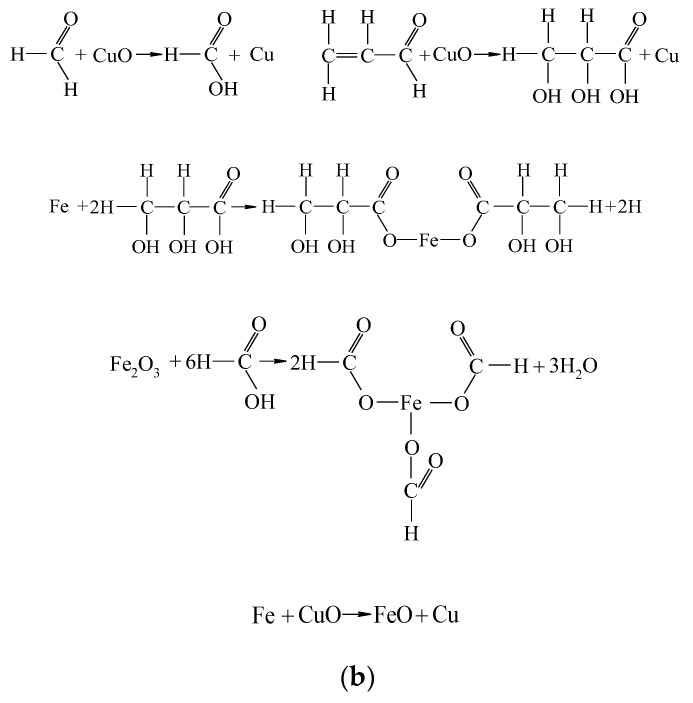
Changes in glycerine during copper coating formation during selective transfer: Stage I (**a**), Stage II (**b**).

Glycerine may undergo the following changes, which may occur during the formation of a copper coating with glycerine lubrication (Figure 7):

- Stage I: glycerine destruction—mechanical and chemical reactions occur, leading to molecular mass reduction.
- Stage II: reduction and dissolution—the impact of reaction products; the reduction in corrosion products and the dissolution of active metals and their compounds.

**Figure 8:**
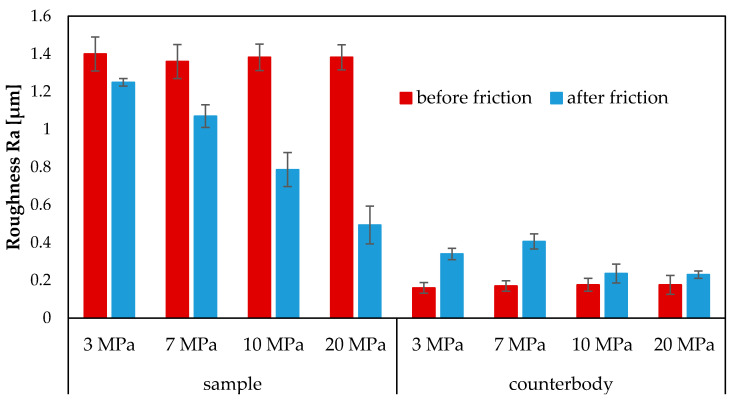
Surface roughness of samples and counterbodies before and after friction under 10 and 20 MPa and with glycerine oil lubrication.

During friction, the surface roughness of the coating decreases. The roughness parameter Ra before friction was about 1.3 µm. However, after the tribological tests, it is 1.25 µm for 3 MPa, 1.07 µm for 7 MPa, 0.787 µm for 10 MPa and 0.493 µm for 20 MPa (Figure 8). In turn, the surface roughness of the counterbody increased slightly. Before friction, the Ra roughness parameter was about 0.18 µm, while after the tribological tests it increased to about 0.23 µm under both 10 MPa and 20 MPa (Figure 8). For a load of 3 MPa it was 0.34 µm and 7 MPa—0.4 µm.

**Figure 9:**
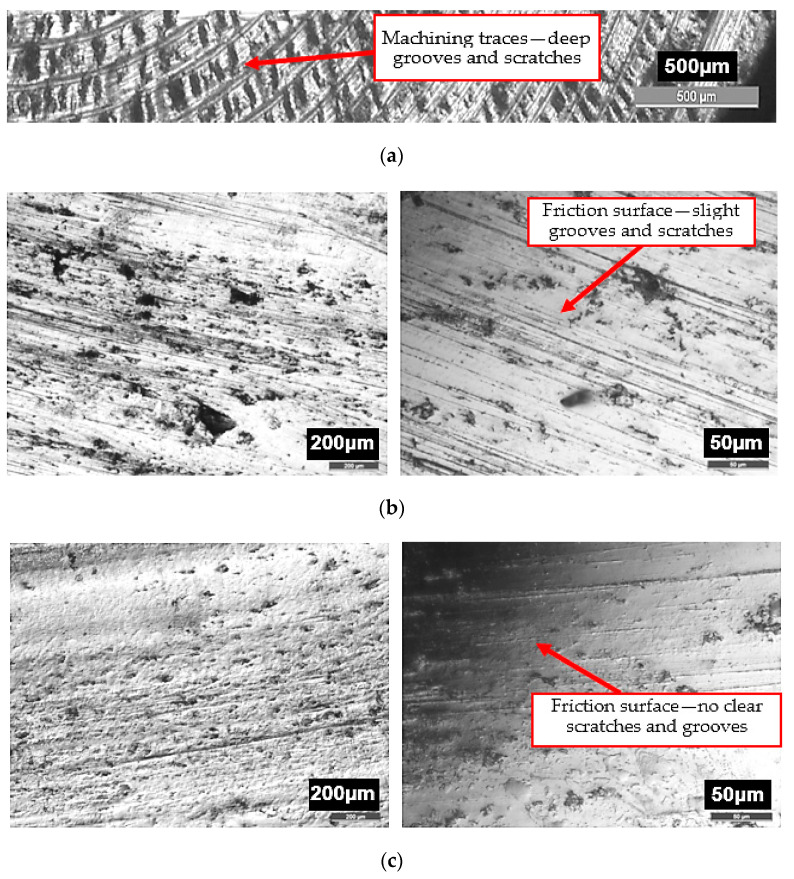
Microscopic images of the coating surface: (**a**) before and after friction under; (**b**) 10 MPa; (**c**) 20 MPa [8] and with glycerine oil lubrication.

The decrease in the friction surface roughness of the coating is confirmed by surface morphology images captured with a metallographic optical microscope. Figure 9 shows the images of the coating surface before and after friction under the loads of 10 and 20 MPa and with glycerine oil lubrication. In the images before friction, one can see machining traces that are removed after friction. Under 10 MPa and 20 MPa, the friction surfaces are largely covered with triboreaction products. Operating surface layers are formed. Traces of friction created on the coating surface lubricated with glycerine oil and loaded with 10 MPa are shown in Figure 9a. There are randomly distributed slight scratches and grooves on the friction surface. Under 20 MPa the grooves and scratches virtually disappear on the surface (the surface is very “smooth”), as shown in Figure 9b. This situation occurs when a soft phase is in a hard matrix [15]. Mn, Ni and Cu all form a soft phase in the analysed coatings. The addition of manganese increases the deformability of iron carbide Fe_3_C by producing manganese iron carbide Fe_0.4_Mn_3.6_C.

**Figure 10:**
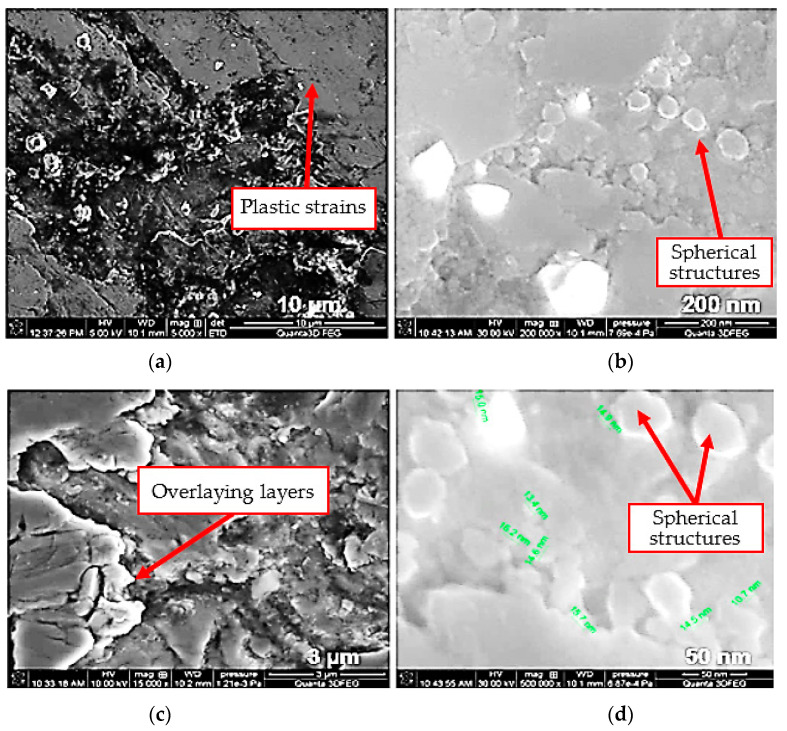
Microscopic images of the coating surface after friction with glycerine oil lubrication and under unit pressure of (**a**,**b**) 10 MPa and (**c**,**d**) 20 MPa [8].

Additionally, SEM images captured at different magnifications show that the friction surface of the coating has a “flaky-laminar” structure (Figure 10a,c). This structure is visible under both 10 MPa and 20 MPa. Formed structures have different shapes and sizes, and in some regions they overlap. According to the literature [35], the operating surface layers with a “flaky-laminar” structure formed during friction protect the mating surfaces against wear. They are produced as a result of tribochemical reactions. However, their formation depends on particular conditions (friction pair materials, lubricants). In the images in Figure 10, one can notice plastic strains as well as stratified secondary structures. In addition to that, one can also observe the presence of spherical structures with the size of 15 nm (Figure 10b,c).

**Figure 11:**
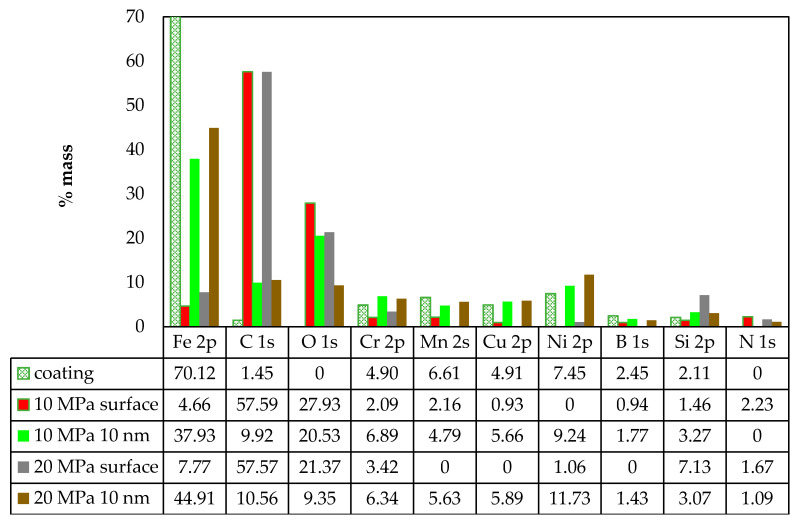
Chemical composition of coating and elemental content on the friction surface and at 10 nm depth under 10 MPa and 20 MPa and with glycerine oil lubrication.

The tribological test results and surface examination reveal that self-organization occurs in the friction pair lubricated with glycerine oil and loaded with 10 and 20 MPa. Friction traces show the presence of products created due to physical and chemical reactions. They cover the mating surfaces to a significant degree. A new OSL (operational surface layer) is formed. Consequently, for further analysis, the operating surface layer was examined by X-ray photoelectron spectroscopy (XPS). Figure 11 gives the elemental content on the coating surface and at the 10 nm depth from the surface measured after the friction process was performed under the loading of 10 MPa and 20 MPa and with glycerine oil lubrication. For comparative purposes, the chemical composition of the coating after hardfacing is given too. The results demonstrate that the iron content increases with depth and load. Under the load of 10 MPa, the iron content on the surface is 4.66% wt. but at the 10 nm depth it drastically increases to 37.93% wt. Under 20 MPa, the iron content on the surface is 7.77% wt. and at 10 nm—it is 44.91% wt. Similar trends can be observed for the contents of chromium, copper, nickel, manganese and boron. This means that their contents increase with depth, but not to such a significant extent as in the case of Fe. The carbon content on the friction surface is similar for 10 MPa and 20 MPa alike. A similar trend can be observed at a depth of 10 nm. In contrast to iron, the carbon content drastically decreases with increasing depth. A considerable carbon content is measured on the friction surface: 57.59% wt. for 10 MPa and 57.57% wt. for 20 MPa, respectively. The oxygen content is similar to that of C, that is—it decreases with depth. The results demonstrate that similar trends can be observed in the elemental composition on the surface and in the surface layer under 10 MPa and 20 MPa.

**Figure 12:**
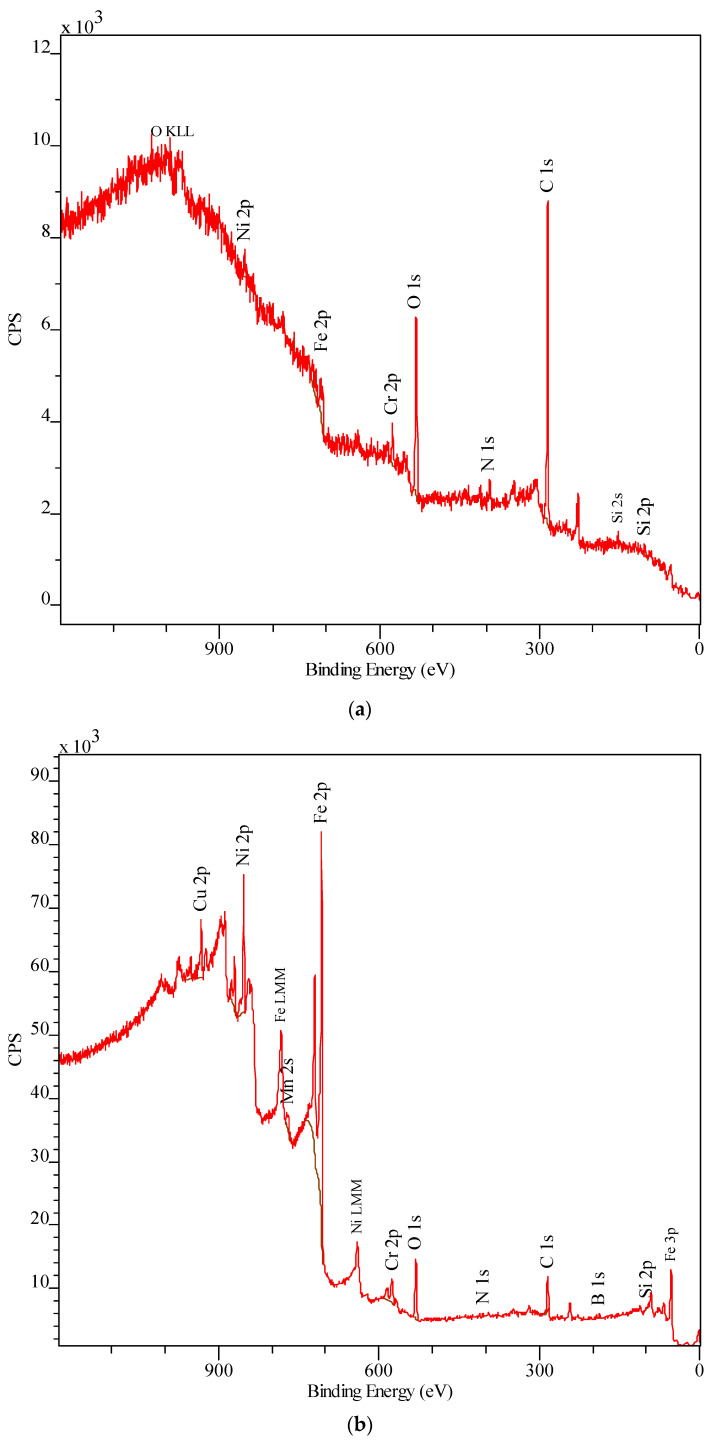

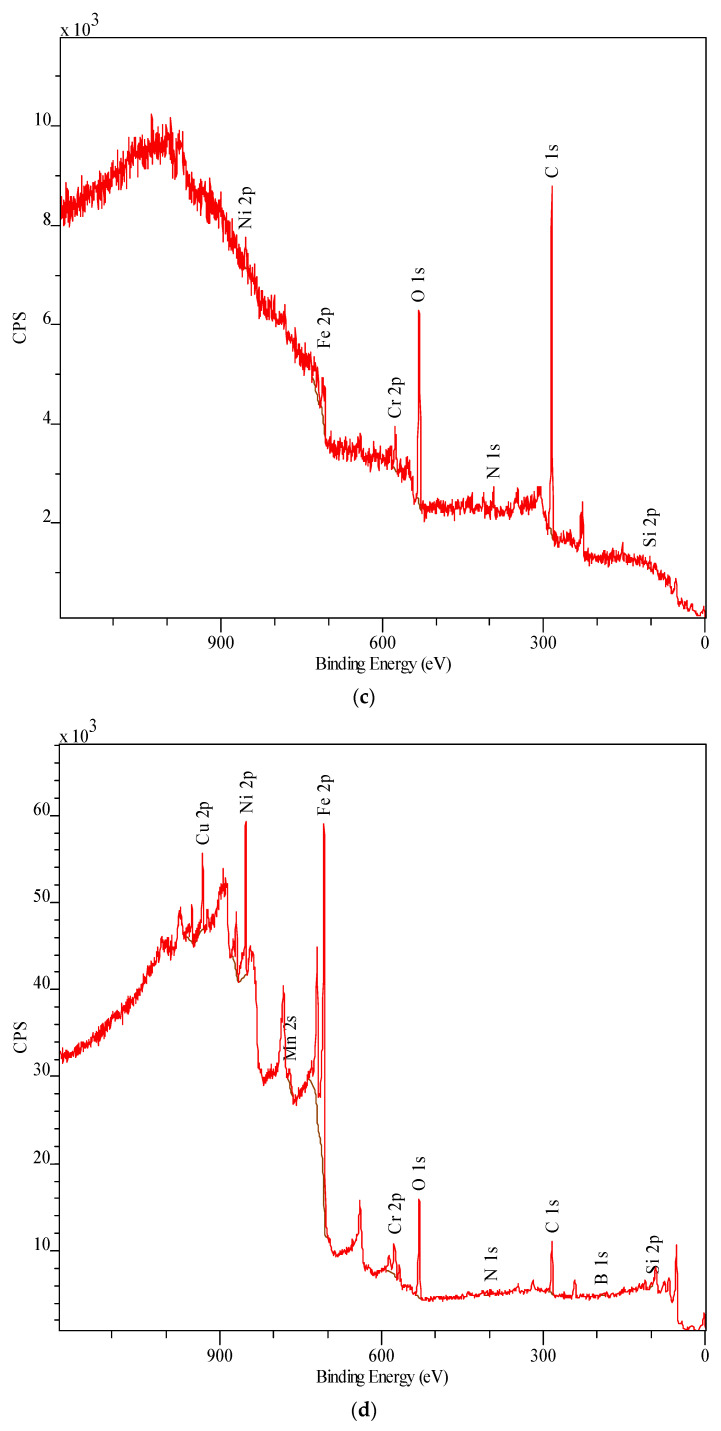
Examples of XPS spectra after friction with glycerine oil lubrication and under unit pressure of (**a**,**b**) 10 MPa and (**c**,**d**) 20 MPa, as analysed (**a**,**c**) on friction trace surface and (**b**,**d**) at 10 nm distance from the surface.

Figure 12 shows examples of XPS spectra that were analysed on the surface of friction traces and at a distance of 10 nm from the surface loaded with 10 MPa and 20 MPa and lubricated with glycerine oil.

The XPS spectra of the surface (Figure 12a,c) significantly differ from others (Figure 12b,d). The surface spectra have no characteristic spectra lines for boron B 1s. There are no visible spectral lines for manganese Mn 2s and copper Cu 2p. It is also worth emphasizing that there are no spectral lines for nitrogen N 1s. No significant differences are observed between the spectra of the samples under 10 MPa and 20 MPa. These spectra were captured at a depth of 10 nm. One can observe characteristic spectral lines of the elements contained in the subeutectic alloy coating as well as the lines of elements such as O and N. Due to the fact that the spectral lines obtained by XPS overlap with the Auger electron spectroscopy lines, their correct interpretation is practically impossible. In the case of the analysed alloy, this takes place for Mn 2s. The forms of the spectral lines of individual elements make it possible to identify the form of a given element in the alloy. Table 3 gives the identified compounds based on individual forms of the elemental lines obtained by XPS.

## 4. Conclusions

The results have demonstrated that the mating of the coatings made of subeutectic alloy Fe-Mn-C-B-Si-Ni-Cr-Cu and the C45 steel counterbody with the use of surface-active lubrication (glycerine oil) leads to self-organization during friction. This trend has been observed during sliding friction for tested loads: 3, 7, 10 MPa and 20 MPa. The tribological results have shown a slight mass loss under 20 MPa (4 mg), 3 and 7 (0.5 mg), and no mass loss under 10 MPa. Under 10 MPa, the mass of the coatings increase by 0.75 mg. The coefficients of friction obtained under 10 MPa and 20 MPa are similar and amount to 0.011. The coefficients of friction obtained under 3 MPa and 7 MPa are also similar and amount to 0.012. The surface examination results have revealed that tribochemical changes occur in the surface layer during friction. The friction surface is covered with triboreaction products. The technological surface layer changes into an operating surface layer with different properties. It has been observed that the quantitative chemical composition of the surface layer changes during friction and that the surface roughness of the coatings is reduced from 1.3 µm to 1.25 µm (3 MPa), 1.07 µm (7 MPa), 0.787 µm (10 MPa) and 0.493 µm (20 MPa). The friction surface of the coatings has a flaky-laminar structure. During friction, various wear-resistant compounds are formed in the operating surface layer. The XPS results have confirmed this trend. These compounds protect the mating surfaces and improve tribological properties of the friction pair. Their compositions and forms change with depth. 

The surface layer of the coating shows the presence of compounds such as oxides (B_2_O_3_, SiO_2_, Cr_2_O_3_, FeO, Fe_3_O_4_, Fe_2_O_3_, CuO, NiO, NO_2_), carbides (Fe_3_C, Cr_7_C_3_), borides (FeB, Fe_2_B, CrB_2_), as well as nitrides (BN, Si_3_N_4_). It can be stated that the friction pair materials (the coating made of Fe-Mn-C-B alloy modified by silicon, nickel, chromium and copper and steel C45) and the surface-active lubricant cause self-organization during friction. The addition of surface-active lubricant brings usable benefits, resulting in self-organization of the surface in the friction process, increasing the operational properties of the tested friction node. Therefore, it is necessary to consciously influence the possibility of selective transfer occurring in the surface layer. The use of Fe-Mn-C-B eutectic alloys for coatings, as well as alloying with various elements and controlling their chemical composition, can significantly affect the self-organization of friction surfaces. It also allows the conscious constitution of the operational features of the friction node.

## Figures and Tables

**Table 1:** Chemical composition of coating and steel C45.

	Element (wt.%)								
	Mn	C	B	Si	Ni	Cr	Mo	Cu	Fe
Coating	6.61	1.45	2.45	2.11	7.45	4.90	-	4.91	Residue
Steel C45	0.5–0.8	0.42–0.5	-	0.1–0.4	max 0.3	max 0.3	max 0.1	max 0.3	Residue

**Table 2:** Physical and chemical properties of glycerine oil.

Physical State	Colour	pH	Melting Point	Boiling Point	Auto-Ignition Point	Flash Point	Absolute Viscosity	Density
			(°C)	(°C)	(°C)	(°C)	(mPa·s)	(g/cm^3^)
liquid	colourless	5	−10°	130	429	177	~150	1.20–1.23

**Table 3:** XPS analysis of individual elements.

B 1s	Cr 2p	Si 2p	Ni 2p	O 1s	Fe 2p	N 1s
boron oxide (B_2_O_3_), borides (FeB, Fe_2_B, CrB_2_)	chromium oxide (Cr_2_O_3_), chromium carbide (Cr_7_C_3_)	metallic form, silicone oxide (SiO_2_)	metallic form, nickel oxide (NiO)	oxides (SiO_2_, NO_2_, CuO, B_2_O_3_, NiO, Cr_2_O_3_, FeO, Fe_3_O_4_, Fe_2_O_3_)	metallic form, iron oxides (FeO, Fe_3_O_4_, Fe_2_O_3_)	nitrides (BN, Si_3_N_4_), nitrogen oxide (NO_2_)
